# Anticarin β Inhibits Human Glioma Progression by Suppressing Cancer Stemness *via* STAT3

**DOI:** 10.3389/fonc.2021.715673

**Published:** 2021-08-02

**Authors:** Min Zhang, Zhi Dai, Xudong Zhao, Gan Wang, Ren Lai

**Affiliations:** ^1^Key Laboratory of Animal Models and Human Disease Mechanisms, Chinese Academy of Sciences/Key Laboratory of Bioactive Peptides of Yunnan Province, Kunming Institute of Zoology - The Chinese University of Hong Kong (KIZ-CUHK) Joint Laboratory of Bioresources and Molecular Research in Common Diseases, National Resource Center for Non-Human Primates, Kunming Primate Research Center, and National Research Facility for Phenotypic & Genetic Analysis of Model Animals (Primate Facility), Kunming Institute of Zoology, Kunming, China; ^2^Kunming College of Life Science, University of Chinese Academy of Sciences, Beijing, China

**Keywords:** natural product, glioma, apoptosis, DNA damage, stemness

## Abstract

Glioma is the most common form of malignant brain cancer. It is very difficult to cure malignant glioma because of the presence of glioma stem cells, which are a barrier to cure, have high tumorigenesis, associated with drug resistance, and responsible for relapse by regulating stemness genes. In this study, our results demonstrated that anticarin β, a natural compound from *Antiaris toxicaria*, can effectively and selectively suppress proliferation and cause apoptosis in glioma cells, which has an IC_50_ that is 100 times lower than that in mouse normal neural stem cells. Importantly, cell sphere formation assay and real time-quantitative analysis reveal that anticarin β inhibits cancer stemness by modulating related stemness gene expression. Additionally, anticarin β induces DNA damage to regulate the oncogene expression of signal transducer and activator of transcription 3 (STAT3), Akt, mitogen-activated protein kinases (MAPKs), and eventually leading to apoptosis. Furthermore, anticarin β effectively inhibits glioma growth and prolongs the lifts pan of tumor-bearing mice without systemic toxicity in the orthotopic xenograft mice model. These results suggest that anticarin β is a promising candidate inhibitor for malignant glioma.

## Introduction

Glioma is the most common and lethal kind of brain tumor, accounting for about 50% of malignant brain tumors. The average survival time from diagnosis to death for glioma patients is less than 15 months, and the 5-year survival rate is about 4.7% (1–4). Subpopulations of cancer cells with high tumorigenic potential and unlimited self-renewal capacity, termed cancer stem cells, were found to have high stemness properties (5–7). It has been demonstrated that these stemness-high malignant cells have high tumorigenic potential, enhanced DNA repair capacity, and resistance to conventional chemotherapies and radiation. Moreover, standard chemotherapy and radiation have been found to induce stemness genes in cancer cells, converting stemness-low cancer cells to stemness-high cancer cells. These stemness cells are likely to survive after therapy and ultimately lead to disease recurrence (7–13). Therefore, developing novel effective therapies for precise targeting stemness-high glioma is urgently needed.

Anticarin β is a natural product isolated from the bark of *Antiaris toxicaria*. Anticarin β is a coumarin derivative with a special structure that has been reported previously (14). The coumarins are an important class of heterocyclic compounds with diverse pharmacological activities and outstanding optical properties. Precious studies have demonstrated that coumarin and its derivatives have a variety of biological activities, including anti-inflammatory, antibacterial, antiviral, and anti-cancer activities which are associated with apoptosis (15–20).

In our study, anticarin β showed attractive bioactive properties to glioma *in vitro* and *in vivo* including selectively inhibit proliferatively and stemness, induce glioma apoptotic and DNA damage, suppress the growth of glioma on mice tumor model. Our finding suggested anticarin β is a potential inhibitor for human glioma.

## Materials And Methods

### Purification and Characterization of Anticarin β

**Figure 1:**
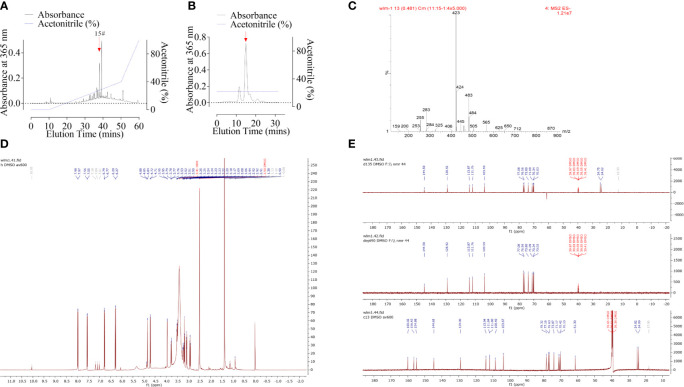
Purification and characterization of anticarin β from *Antiaris toxicaria*. **(A)** Crude extract containing anticarin β was separated by a C_18_ column and absorbance at 365 nm was recorded. **(B)** Fractions containing anticarin β (indicated by arrow in A) was further purified by a C_18_ reverse phase (HPLC) column and the anticarin β was indicated by a red arrow. **(C–E)** Anticarin β was characterized by ESI-MS spectrum **(C)**, ^1^H-NMR spectrum (DMSO, D), and ^13^C DEPT NMR spectrum (DMSO, E).

The powdered bark of *Antiaris toxicaria* was socked in EtOH and concentrated to give a crude extract after removal of the solvents. The crude extract was extracted successively with petroleum ether, dichloromethane, ethyl acetate, and n-butanol. The n-butanol phase extract exhibited a significant cytotoxic activity on glioma cells. We then performed column chromatography on the crude extract, using Macro-porous resin column, Medium Pressure Liquid Chromatography (MPLC), and High-Performance Liquid Chromatography (HPLC). The extract was divided into several peaks by C_18_ column according to absorbance at 365 nm (**Figure 1A**). Peak 15# (indicated by arrow) was further purified by a C_18_ reverse-phase (HPLC) (**Figure 1B**) column and identified by ESI-MS spectrum (**Figure 1C**), ^1^H NMR spectrum (DMSO, **Figure 1D**), and ^13^C DEPT NMR spectrum (DMSO, **Figure 1E**). The compound, anticarin β, is consistent with previous reports (14).

### Cells and Culture

All human cell lines were obtained from the Conservation Genetics CAS Kunming Cell Bank (Kunming, Yunnan, China), which performs authentication on its cell lines. Human cell lines were cultured in DMEM/F12 supplemented with 10% heat-inactivated FBS, 100 U/mL penicillin, and 100 μg/mL streptomycin at 37˚C and 5% CO_2_ in a humidified incubator.

Normal mouse neural stem cell line (mNSC) was obtained from Sigma-Aldrich (Darmstadt, Germany). Neural stem cells were cultured in glioma stem cell medium that consisted of DMEM/F12, 1 x B27, 50 ng/mL bFGF, and 50 ng/mL EGF supplemented with 100 U/mL penicillin and 100 µg/mL streptomycin. Culture dishes were pre-coated with 10 µg/mL laminin for 8 h at 37˚C in a humidified incubator before neural stem cells were seeded and maintained at 37˚C in a humidified incubator with 5% CO_2_.

### Morphological Observation

The morphology of 10 µM anticarin β-treated cell lines (U87-MG, T98G, and mNSC) at 24 h after treatment was examined and images were observed by phase-contrast microscopy (magnification, 40x).

### MTS Assay

A total of 2 x 10^4^ cell lines (U87-MG, T98G, and mNSC) in 150 µl per well were seeded into 96-well plates and treated with 50 µl anticarin β at 10 μM, 5 μM, 2.5 μM, 1.25 μM, 625 nM, 312.5 nM, 156.25 nM, 78.125 nM, 39.0625 nM, respectively. Cells treated with the same volume of phosphate buffer saline (PBS, 0.01 M) were used as normal control. All groups of cells were incubated for 24 h at 37˚C with 5% CO_2_ after treatment with anticarin β. MTS reagent was diluted 1:10 with fresh complete medium and added 100 μL per well. After incubating for 1 hour, the absorbance was measured at 490 nm. The inhibition of cells treated with anticarin β was compared with the control group and expressed as a percentage. The half-maximal inhibitory concentration (IC_50_) was calculated using GraphPad Prism 6.0 (GraphPad Software).

### Colony Formation Analysis

Human glioma cells, U87-MG and T98G, were plated in 6-well plates (100 cells each well) and incubated for 7 days with treatments of anticarin β (0.5 μM). The control groups were treated with the same volume of PBS. The cells were then fixed with 4% formaldehyde for 20 min and stained with 0.25% crystal violet for 15 min. The number of colonies, defined as > 50 cells/colony, was counted and quantified. The data were analyzed using ImageJ.

### Cell Sphere Formation Assay

3.2% soft agar was made by 0.4 g low gelling agar, mixed with 13 mL water, and sterilized by high-pressure steam sterilization. Then, 3.2% soft agar was diluted to 0.8% with medium and plated 1 mL per well in a 6-well plate as the bottom layer of agar. After colling at room temperature, 2 x 10^4^ cells were mixed with 0.8% soft agar to 0.4% and plated 1 mL per well in a 6-well plate, cooled, and added 1 mL medium each well. The plate was cultured in a humidified incubator at 37˚C with 5% CO_2_ and the medium was supplemented every 3 days. After the clonal spheres had reached 20 µm, they were treated with 1 μM anticarin β or the same volume of PBS every 3 days. After two weeks, the clonal spheres were collected, washed, and fixed with 4% paraformaldehyde containing 0.005% crystal violet for 2 h at room temperature. Images were obtained and the clonal spheres were counted.

### Transwell Migration and Invasion Assays

Cell migration and invasion abilities were detected *via* Transwell assay (8-μm filter). For the migration assay, 5 x 10^4^ cells resuspended in 200 μL culture medium with treatments of anticarin β (0.5 μM) without FBS were inoculated into the top chambers, the control groups were treated with the same volume of PBS; the bottom chambers were supplemented with 500 μL medium supplemented with 10% FBS. After 48 h, the non-migrating cells were removed, and the cells migrating through the membrane were fixed with methanol for 30 min and stained with 0.1% crystal violet for 15 min. The migrating cells were counted and the data were analyzed using GraphPad Prism 6.0 (GraphPad Software). For the invasion assay, the same number of cells was seeded into the top chambers that were precoated with Matrigel solution (BD Diagnostics), and the following experimental steps were the same as the migration assay.

### Cell Proliferation Assay

Cell proliferation assay was performed using EdU Cell Proliferation Kit with Alexa Fluor 594 (Beyotime, Shanghai, China) and following the protocol described by the manufacturer. Cells were seeded onto a 24-well plate with coverslips, with 1x10^5^ cells/per well. Anticarin β (0.5 μM) was added and the control wells were treated with the same volume of PBS. Cells were treated for 12 h at 37˚C in a humidified incubator. Then, cells were treated with EdU (10 µM) for 2 h at 37˚C in a humidified incubator with 5% CO_2_. Following incubation, the cells were fixed in 4% paraformaldehyde, permeabilized with 0.5% Triton X-100 in PBS containing 3% BSA (PBS - BSA) for 20 min. Then, the cells were immersed in Click reaction mixture for 30 min and in Hoechst 33324 (10 mg/mL; diluted 1:1,000 in PBS) for 10 min at room temperature protected from light. Before the observation, the cells were washed twice with PBS and mounted onto a glass slide. Images were obtained using a fluorescent microscope at the magnifications 20x.

### Real Time-Quantitative Analysis

The total RNA extraction of cells was performed using Trizol reagent (Invitrogen; Thermo Fisher Scientific, Inc.). 1 μg of total RNA was reverse transcribed using FastKing gDNA Dispelling RT SuperMix (Tiangen Biotech). The action was carried out at 42°C for 15 min and terminated by deactivation of the enzyme at 95°C for 5 min. PCR was conducted using EvaGreen 2X qPCR MasterMix (ABM, BC, Canada) in StepOne™ Real-Time PCR System (ThermoFish Scientific, MA, USA) using the manufacturer’s default settings for relative quantification of standard Ct. Baseline and threshold values were automatically detected using the StepOne™ Software v2.3 (ThermoFish Scientific, MA, USA) with default settings. Gene expression was calculated using the comparative ΔΔCT method with the housekeeping genes *GAPDH* for normalization. The primers used are provided as follows. *Oct4*: forward: 5’-TAG TCC CTT CGC AAG CCC T-3’, Reverse: 5’-CGA GAA GGC GAA ATC CGA AG-3’; *Nanog*: forward: 5’-CAA TGG TGT GAC GCA GGG AT-3’, Reverse: 5’-GGA CTG GAT GTT CTG GGT CTG-3’; *Klf4*: forward: 5’-ATG CTC ACC CCA CCT TCT TC-3’, Reverse: 5’-TTC TCA CCT GTG TGG GTT CG-3’; *Sox2*: forward: 5’-ACC AGC GCA TGG ACA GTT AC-3’, Reverse: 5’-CCG TTC ATG TAG GTC TGC GA-3’; *Stat3*: forward: 5’-ACC ATT GAC CTG CCG ATG TC-3’, Reverse: 5’-TAG GCG CCT CAG TCG TAT CT-3’; *Cd133*: forward: 5’-AGT CGG AAA CTG GCA GAT AGC-3’, Reverse: 5’-GGT AGT GTT GTA CTG GGC CAA T-3’; *GAPDH*: forward: 5’-GAA AGC CTG CCG GTG ACT AA-3’, Reverse: 5’-GCA TCA CCC GGA GGA GAA AT-3’.

### Neutral Comet Assay

DNA damage was assessed by single-cell gel electrophoresis assay under neutral conditions as described (21). Human glioma cell lines U87-MG treated with anticarin β (1 μM) for 12 hours were suspend at 2 x 10^5^ cells/mL in PBS. Combine the cell suspension with 1% molten low melting point agarose and immediately pipette it onto a slide. After placing slides at 4°C for 10 min, immerse slides in lysis solution overnight. Then, slides were immersed in freshly Neutral electrophoresis solution (pH = 9.0) for 30 min. Electrophoresis was carried out at the rate of 1.0 V/cm for 45 min at 4°C. After electrophoresis, the slides were immersed with DNA precipitation solution for 30 min and ice-cold 70% ethanol for 30 min. Subsequently, the slides were dried at room temperature, and DNA was stained with 50 μL of SYBR Green I dye [MCE, 1:10,000 in Tris-EDTA buffer (pH 7.4)] for 15 min and immediately analyzed using a fluorescence microscope. Data were analyzed using CometScore (TriTek, Sumerduck, VA).

### Apoptosis Assay

Apoptosis assay was performed using GreenNuc™ Caspase-3 Assay Kit for Live Cells (Beyotime, Shanghai, China) and fluorescent terminal deoxynucleotidyl transferase dUTP nick end labeling (TUNEL) assay for tissue sections.

For cells, human glioma cell lines U87-MG (1 x 10^5^ cells/well) were plated in a 6 cm glass-bottom dish. Cells were treated with 1 μM anticarin β for 24 h. Each dish was incubated with 5 μM GreenNuc™ Caspase-3 Substrate and Hoechst 33324 (10 mg/mL; diluted 1:1,000 in PBS) for 30 min in dark at room temperature. Before observing, the dishes were washed with PBS. Images were obtained using a fluorescent microscope at the magnification of 20x and 40x.

For tissue sections, Sections were de-paraffinized and rehydrated by incubation in a xylene and ethanol gradient. Antigen retrieval was performed in sodium citrate buffer (pH 6.0) at 95°C for 5 min. Tunel assay was performed using TUNEL FITC Apoptosis Detection Kit (Vazyme, Nanjing, China) according to the manufacturer’s protocol. The nuclei were counterstained with DAPI for 5 min. Images were obtained using a fluorescent microscope, and the staining was scored using ImageJ.

### Western Blot Analysis

Human glioma cell lines U87-MG, treated with or without 1 μM anticarin β for a different time, and tumor tissues were collected and lysed in RIPA buffer (Sigma, St. Louis, MO), containing Protease Inhibitor Cocktail (MCE, Shanghai, China) and Phosphatase Inhibitor Cocktail II (MCE, Shanghai, China). Lysates were sonicated, clarified by centrifugation, and protein concentrations were measured using the BCA protein assay (Biosharp, Hefei, China). Proteins were separated on 10% acrylamide SDS–PAGE gels, transferred to PVDF membranes. After blocking, the membranes were incubated with primary antibodies at 4°C overnight and then incubated with secondary antibodies at room temperature for 1 h. The signal detection was performed using enhanced chemiluminescence ECL substrate (Bio-Rad, Hercules, USA). Band images were acquired using a LAS 4000 mini system (GE Healthcare, Little Chalfont, England). Each experiment was independently repeated in triplicate. The antibodies used as followed: Primary antibodies Phospho-p38 (Thr180/Tyr182) (4511, CST), p38 (8690, CST), phospho-Erk1/2 (Thr202/Tyr204) (9101, CST), Erk1/2 (4695, CST), phospho-JNK (Thr183/Tyr185) (9255, CST), JNK (9252, CST), phospho-Akt (Ser473) (4060, CST),Akt (pan)(4691, CST), p-STAT3 (Ser727) (9134, CST), STAT3 (12640, CST), E-cadherin (14472, CST), N-cadherin (13116, CST), β-catenin (8480, CST), vimentin (5741, CST), CD44 (5640, CST), Nanog (4903, CST), Sox2 (3579, CST), Oct4 (2840, CST), CD133 (64326, CST), Bcl-2 (15071, CST), Bax (89477, CST), clv-caspase3 (9661, CST), clv-PARP (5625, CST), GAPDH (97166, CST); secondary goat anti-mouse HRP-IgG (7076, CST) and goat anti-rabbit HRP-IgG (7074, CST) antibodies were used for immunoblotting.

### Animal Experiment

Male NOD/SCID mice (5 weeks old, 15-18 g, N = 18) were purchased from Beijing Vital River Laboratory Animal Technology Company. Before the experiments, mice were performed to adapt to the environment for a week. A total of 18 male NOD/SCID mice were injected with 2 x 10^5^ U87-MG cells in the right hippocampus. The injection position of each mouse was relatively consistent, and the injection depth was 3 mm. 3 days post-inject, the mice were randomly divided into three groups (6 mice per group). The intracranial administration of PBS or anticarin β (1 mg/kg) using Alzet Osmotic Pumps was started on animals each day, and the intragastric administration of temozolomide (TMZ, 50 mg/kg) was started on animals once. All mice were used *in vivo* research after death. The critical organs (heart, liver, spleen, lungs, and kidneys) and brains were harvested. The brain is divided into two parts according to the location of injection, and the tumor area was measured. Half of tissues were formalin-fixed, cut into small pieces, paraffin-embedded, sectioned, and stained with haematoxylin & eosin (H&E) or antibodies. The effect of anticarin β was evaluated on specimens in at least three different sections. Half of tissues were frozen at -80°C for other experiments.

### Cell Detection

Flow cytometry was used to analyze the percentage of cancer stem cells in T98G and U87-MG. Tumor cells were collected, rinsed, and fixed in 4% polyoxymethylene for 30 min. Then, cells were incubated with anti-CD44 antibodies for 60 min at room temperature in the dark. After washing with PBS, cells were incubated with FITC-labled secondary antibodies for 60 min at room temperature in the dark. The cells were resuspended in PBS and analyzed using an LSRFortessa flow cytometer (BD, San Jose, CA, USA). The data were analyzed using FlowJo software (Beckman Coulter). Each experiment was carried out in triplicates.

### Statistical Analysis

The quantitative data are represented as the mean ± s.d. of triplicate experiments. The difference between two groups was compared with Student’s *t*-test. Ordinary one-way ANOVA with multiple comparisons tests was used for examining differences among multiple groups. The overall survival of mice was analyzed by the Kaplan-Meier method and compared with the Log-rank test. All statistical analyses were performed using GraphPad Prism 6.0 (GraphPad Software), and p-values were calculated, significance is represented as follows, * (*P* < 0.05), ** (*P* < 0.01), and *** (*P* < 0.001).

## Results

### Anticarin β Selectively Inhibits the Proliferation of Human Glioma Cells

**Figure 2:**
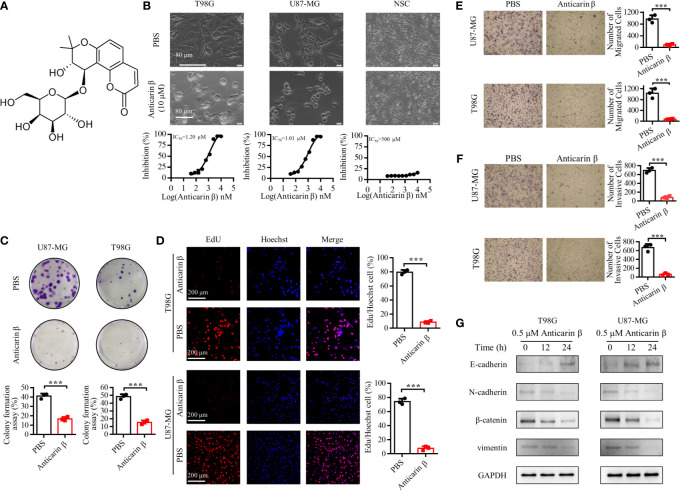
Anticarin β inhibits the proliferation of human glioma cells. **(A)** The chemical structure of anticarin β. **(B)** Morphological changes and dose-dependent cytotoxicity on human glioma cell lines (U87-MG and T98G). Cells were treated with 0-10 μM anticarin β for 24h and then observed by phase-contrast microscopy. Cell viability was calculated using MTS analysis. **(C)** The colony formation results of U87-MG and T98G after treatments of anticarin β (0.5 μM) for 7 days were exhibited. **(D)** The number of proliferative cells labeled by EdU (red) and cell nucleus labeled by Hoechst 33324 (blue) was different between anticarin β (0.5 μM, 12 hours) and PBS group in U87-MG and T98G. Scale bar, 200 μm. **(E)** Migration and **(F)** invasion ability of human glioma cell lines U87-MG and T98G treated with anticarin β (0.5 μM) for 48 hours were measured with transwell migration assay and transwell invasion assay. **(G)** Western blotting analysis of E-cadherin, N-cadherin, β-catenin, vimentin in T98G and U87-MG treated with or without anticarin β (0.5 μM). Data are presented as mean ± s.d. of three independent experiments conducted in duplicate, ***P < 0.001 *versus* the control group.

A function-based purification strategy was guided the potential anti-glioma compound identification in *Antiaris toxicaria*. Anticarin β was purified and characterized (**Figure 1**). The structure of anticarin β was established as 4’-β-glucosyl-khellactone, angular dihydropyrano- coumarin, shown in **Figure 2A**. Then, the proliferation-inhibiting efficacy of anticarin β against human glioma cells (U87-MG and T98G) and normal mouse neural stem cells (mNSCs) was evaluated. Anticarin β effectively inhibited the proliferation of glioma cells with concentration-dependent manner and no significant cytotoxicity was observed in normal mouse neural stem cells (**Figure 2B**). Anticarin β effectively inhibits human glioma cells (U87-MG and T98G) with half-maximal inhibitory concentration (IC_50_) ~1.01 ± 0.10 and 1.20 ± 0.11 μM at 24 h, respectively. For the mNSCs, the IC_50_ value was more than 500 μM. Furthermore, the colony formation assay was performed to determine the effect of anticarin β on cell proliferative potential of human glioma cells. The colonies formed after anticarin β treatment was reduced 25% in U87-MG and 35% in T98G compare with the control (**Figure 2C**). As shown in **Figure 2D**, EdU-positive proliferative cells decreased by more than 70% in cells treated with anticarin β compared with PBS group. Anticarin β had no significant inhibitory effect on proliferation in mNSCs (**Figure S1A**). This result indicated that anticarin β could suppress proliferation by reducing DNA synthesis. Additionally, transwell cell migration, matrigel invasion assay, and western blot analysis showed that the treatment of anticarin β can suppress cell migration and invasion in both human glioma cell lines U87-MG and T98G compared to control (**Figures 2E–G**), and has no effects on migration and invasion in mNSC (**Figures S1B, C**). These results above illustrated that anticarin β selectively inhibit the human glioma cells and has no toxic to normal neural stem cells, suggesting anticarin β may targeting tumor-specific pathways.

### Anticarin β Inhibits the Stemness of Human Glioma Cells

**Figure 3:**
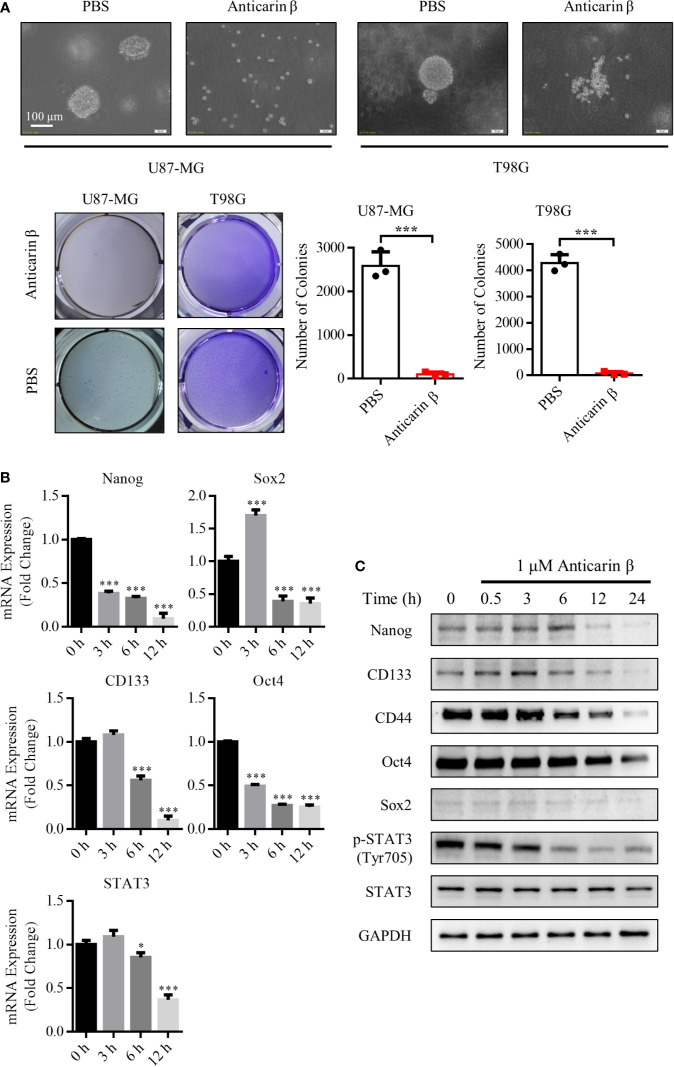
Anticarin β inhibits the stemness of human glioma cells. **(A)** Human glioma cell lines U87-MG and T98G clonal sphere morphology were damaged following treatment with anticarin β (1 μM). Cells sphere formation was stained with 4% paraformaldehyde and 0.005% crystal violet for 2 hours. The quantitative data of the number of clonal spheres were shown. **(B)** The mRNA expression of the stemness-related genes was measured in U87-MG cells with anticarin β (1 μM) for different times using RT-qPCR analysis. **(C)** The expression of the stemness-related proteins and CD44 was detected by Western Blotting in U87-MG cells after treated with anticarin β (1 μM) for different times. Data are presented as mean ± s.d. of three independent experiments conducted in duplicate, *P < 0.05 and ***P < 0.001 *versus* the control group.

The stemness of cancer plays an essential role in tumorigenic, drug resistance, and relapse. The stem cells contain self-renewal capacity and give rise to the various cell lineages that comprise the tumor; they have been postulated as responsible for recurrence, and drug resistance of glioma (5). Thus, here we attempted to explore the effects of anticarin β on the stemness of glioma. CD44 have been identified as cancer stem cell markers for isolating and enriching cancer stem cells (22). Flow cytometric analysis showed that the high expression of CD44 in T98G and U87-MG. These results indicated that the high percentage of cancer stem cells in the T98G and U87-MG (**Figure S2A**). We examined whether anticarin β affects the self-renewal of glioma through cell sphere formation assay. U87-MG and T98G were treated with 1 μM anticarin β, clonal spheres were damaged, and the number of clonal spheres decreased dramatically compared with control (**Figure 3A**). Further, the expression of several stemness-associated factors and markers was examined in U87-MG with anticarin β (1 μM) for the different times by RT-qPCR analysis and Western Blotting analysis (**Figures 3B, C**). The treatment of U87-MG with anticarin β significantly decreased mRNA expression of many stemness factors and markers, including *Nanog, Oct4, Sox2, Cd133, and Stat3*. The protein expression of CD44, CD133, Nanog, Oct4, Sox2, and p-STAT3(Tyr705) was suppressed by anticarin β treatment for increasing time. Taken together, these results demonstrate that anticarin β destroy the self-renewal capacity of glioma cancer stem cells.

### Anticarin β Induces Apoptosis and DNA Damage in Human Glioma Cells

**Figure 4:**
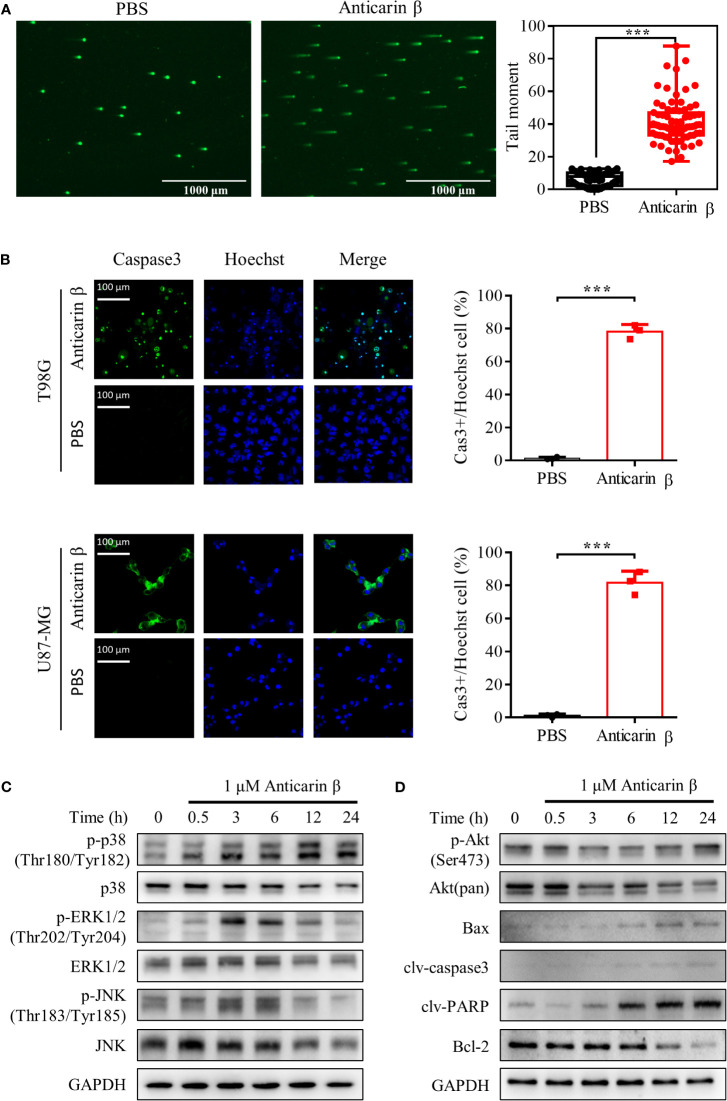
Anticarin β induces apoptosis by DNA damage in human glioma cells. **(A)** The DNA damage in human glioma cell lines U87-MG treated with anticarin β (1 μM) for 12 hours was measured using the neutral comet assay. Scale bar, 1000 μm. **(B)** U87-MG and T98G cells were treated with anticarin β (1 μM) or PBS for 24 hours, followed by fluorescence analysis of caspase 3 probe (green) and Hoechst 33324 (blue). Scale bar, 100 μm. **(C, D)** Western blotting analysis showed the time-dependent effects of anticarin β (1 μM) in U87-MG cells on Akt, MAPKs signal pathways, and apoptotic-related proteins. Data are presented as mean ± s.d. of three independent experiments conducted in duplicate, ***P < 0.001 *versus* the control group.

In cancer therapies, DNA damage is a countermeasure to control cancer cell fate, death, or survival, to limit cancer progression. Upon DNA damage, cells initiate several response pathways to activate DNA repair or apoptosis (23). Previous experiments have shown that anticarin β can suppress DNA synthesis. For this reason, to further explore the mechanism of anticarin β’s cytotoxicity, we performed Comet Assay to assess DNA damage. Anticarin β treatment could lead to obvious DNA damage (**Figure 4A**). Then, the caspase3 probe fluorescence analysis indicated that anticarin β (1 μM, 24 h) induced caspase-3 activation in human glioma cells and suppressing glioma cell growth (**Figure 4B**). This data indicated that anticarin β-induced cell death was, at least partly, associated with apoptosis induction.

To further explore the underlying molecular mechanism, western blot analysis was used to investigate signaling pathways. Akt, p38, extracellular-signal-regulated kinase (ERK), c-Jun N-terminal kinase (JNK), Bax, B-cell lymphoma-2 (Bcl-2), caspase3, and PARP both play important roles in regulating cancer cell proliferation and apoptosis (24, 25). Therefore, related proteins were examined in anticarin β-treated U87-MG cells by Western blotting (**Figures 4C, D**). Anticarin β treatment caused activation of p-p38 (Thr180/Tyr182), Bax, caspase3, and PARP. p-JNK (Thr183/Tyr18), and p-ERK1/2 (Thr202/Tyr204) were activated from 3 to 12 h then decreased. p-Akt (Ser473), and Bcl-2 was suppressed. These results elucidated that anticarin β inhibits cell growth and induces apoptosis in U87-MG cells *via* affecting Akt, MAPKs pathways, and apoptotic-related proteins.

### Anticarin β Inhibits the Glioma Growth in Orthotopic Xenograft Mice Model

**Figure 5:**
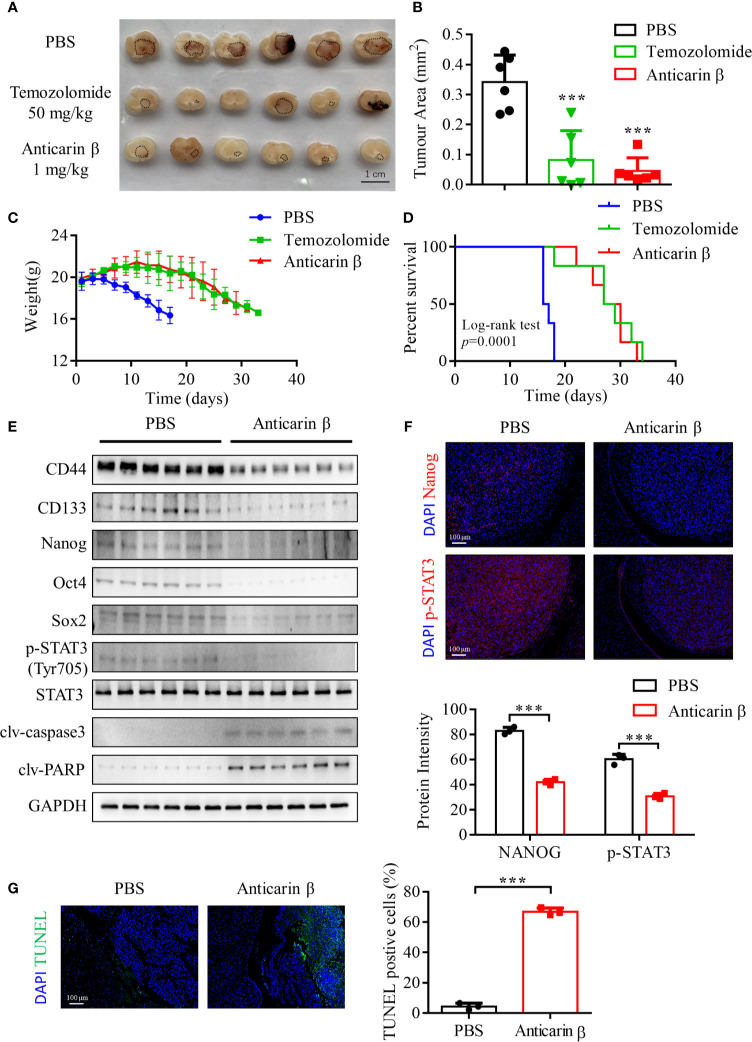
Anticarin β inhibits the tumor growth of the human glioma orthotopic xenograft *in vivo*. U87-MG (2 x 10^5^) cells were inoculated in the right hippocampus of NOD/SCID mice. 3 days post inject, the intracranial administration of PBS or anticarin β (1 mg/kg) using Alzet Osmotic Pumps was started on animals each day, and the intragastric administration of temozolomide (TMZ) (50 mg/kg) was started on animals once. All mice were used *in vivo* research after death. The tumor area was measured by brain sections, tumor areas are highlighted in black circles on the brain sections **(A, B)**, and the comparison of weight and Kaplan-Meier curves among each group is presented **(C, D)**. **(E)** Tumor samples were collected, and protein was extracted for western blot analysis. Stemness-related proteins and apoptosis proteins were detected, with GAPDH used as control. **(F)** Tumors were subjected to immunofluorescent stain and the level of Nanog and p-STAT3 was evaluated. Cells were stained for the DAPI (blue), Nanog (red), p-STAT3 (red). Scale bar, 100 μm. **(G)** Tumors were subjected to TUNEL assay and the percentage of TUNEL-positive cells were calculated. Data represent mean ± s.d. N = 6. ***P < 0.001 *versus* the Control group.

We next tested whether anticarin β can suppress glioma growth *in vivo* using an orthotopic xenograft NOD/SCID mice tumor model. Human glioma cell line U87-MG (2 x 10^5^) was injected into the right hippocampus of mice. Mice were randomly divided into 3 groups (negative group (PBS), positive group (temozolomide, the most commonly used alkylating agent in glioma chemotherapy), and anticarin β group. Consistent with our *in vitro* results, anticarin β significantly suppressed the growth of glioma cells (**Figures 5A, B**). Mice weight has no significant change until ~23 days after glioma cell injected, and the survival of tumor-bearing mice has been prolonged in anticarin β group (**Figure 5C, D**). There was no obvious toxicity in the major organs of mice treated with anticarin β (**Figure S3A**). Tumor samples were collected, and western blot analysis showed that the expression of stemness-related proteins and apoptosis proteins *in vivo* was similar to that *in vitro*. In anticarin β-treated tumor tissues, CD44, CD133, Nanog, Oct4, Sox2, p-STAT3 were suppressed, and caspase3, PARP was activated (**Figure 5E**). Furthermore, Nanog and phosphorylated STAT3 in anticarin β-treated tumor tissues were decreased by immunofluorescent stain (**Figure 5F**). TUNEL staining confirmed a higher percentage of apoptotic cells in brain tumors treated with anticarin β than PBS (**Figure 5G**). Our data suggested that anticarin β block tumorigenesis, prolong overall survival duration, and has no obvious systemic toxicity *in vivo*, thus highlighting the potential of anticarin β to develop an inhibitor for glioma in the future.

## Discussion

The current standard therapeutic strategy for glioma is mainly relied on surgical treatment, followed by radiotherapy and chemotherapy. The limitations of surgical treatment make it impossible to completely remove tumors, and glioma cells are often tolerant to chemotherapy and radiotherapy. The prognosis for patients is still poor with a low five-year survival rate (1–4). Preciously, some novel strategies of therapy have been investigated. Many chemotherapeutic agents isolated from natural plants are served as the effective drug of cancer cells (13, 26–28).

Anticarin β, a coumarin derivative isolated from the bark of *Antiaris toxicaria Lesch*, has been reported to have potential anticancer activities (14). However, the molecular mechanisms underlying anticarin β-mediated suppression of tumors remain poorly understood. In this study, we determined the anticancer effector and associated mechanisms of anticarin β on human glioma cell lines *in vitro*. With the concentrations used in our study, anticarin β effectively suppressed the proliferation of human glioma cell lines (U87-MG and T98G) in a dose-dependent manner and showed no significant cytotoxicity to mouse neural stem cells. Additionally, our study indicated that anticarin β could suppress the migration, invasion, and proliferation of human glioma. *In vivo*, anticarin β had no obvious systemic toxicity, dramatically reduced tumor growth, and prolong overall survival duration. However, as for its specific adverse effects on mice, further study is still necessary for the future.

Stemness, initially defined by the expression of stem cell genes, is a property shared by embryonic stem cells and adult stem cells (7, 29). Glioma stem cell markers differentially expressed on glioma stem cells and functionally associated with the maintenance of glioma stem cells are ideal targets for glioma stem cells (6, 30). CD133, one of the earliest stem-cell surface markers, is essential for glioma stem cell maintenance and neurosphere formation. It is also an indicator of resistance to conventional therapy (31). CD44 is cancer stem cell marker and critical player in regulating self-renewal, tumor initiation, metastasis, and chemoradio-resistance (22). Some transcriptional factors with well-recognized functions in embryonic development, such as Nanog, Oct4, Sox2, STAT3, have been identified as oncogenic drivers which can affect the fate of glioma stem cells and regulation of glioma development (32). In addition to different gene expressions, stemness can be measured by a cell’s ability to form spheres (33). In our study, we found that anticarin β inhibits clonal spheres forming in human glioma cells and reduces the expression of stemness-related markers, such as Nanog, Oct4, Sox2, CD133, STAT3, and CD44. Besides, anticarin β suppresses stemness-related protein expression in tumor model. These data indicated that anticarin β could inhibit cancer stemness by modulating the expression of several stemness-related genes.

Apoptosis is modulated by death receptors and death factors, to control cell proliferation and a reaction to cell injury. Tumorigenesis includes the occurrence of an imbalance between cell proliferation and apoptotic cell death, leading to the escape of apoptosis and over-proliferation. DNA damage can result in genomic instability, apoptosis, cell cycle checkpoint alteration, or postmitotic death (1, 34, 35). Signaling through MAPKs, Akt, STAT3 are known to play important role in various cell functions in gliomas, such as cell proliferation, angiogenesis, apoptosis, inflammation, oncogenesis, and differentiation (25, 36–43). Previous studies have shown that STAT3 promotes cell proliferation and inhibits apoptosis by increasing the expression of oncogenes, including survivin, c-Myc, Bcl-2 (44). The pro- or anti- apoptotic effects of MAPK activation depend on the duration of the signal. Persistent activation of JNK, p38, and ERK1/2 induce apoptosis (24, 25, 45, 46). In this study, our data demonstrated that anticarin β can induce DNA damage and apoptosis in human glioma cells. Western blotting results showed that anticarin β decreased the levels of Bcl-2, and increased the levels of Bax, clv-capase3, and clv-PARP, which indicated apoptosis-induced. Besides, anticarin β significantly decreased the phosphorylation levels of Akt, STAT3, and cause prolonged JNK, p38, ERK1/2 activation. These results demonstrated that the functional importance of the Akt, STAT3, and MAPKs pathways in anticarin β-induced DNA damage and apoptosis in human glioma cells.

In conclusion, our findings above suggested that anticarin β effectively and selectively suppress the growth, proliferation, and stemness of human glioma cells by inducing apoptosis and DNA damage, which likely through modulation of STAT3, Akt, MAPKs pathways, and apoptotic-related proteins. In the human glioma orthotopic xenograft models, administration of anticarin β exhibited obvious anti-cancer activity. Furthermore, anticarin β with low cytotoxicity has the potential to be a promising compound to prevent human glioma tumorigenesis. The anti-tumor effect of anticarin β against glioma could be combined with other FDA-approved DNA-damaging anti-cancer drugs in future studies.

## Data Availability Statement

The raw data supporting the conclusions of this article will be made available by the authors, without undue reservation. The original data is available at Mendeley Data (https://doi.org/10.17632/fhw7ff6gd3.1).

## Ethics Statement

The animal study was reviewed and approved by Committee of Kunming Institute of Zoology, Chinese Academy of Sciences (authorization number: SMKX-2021-03-013).

## Author Contributions

Conception and design: MZ, GW, and RL. Development of methodology: MZ, XZ, GW, and RL. Acquisition of data (provided animals, provided facilities, etc.): MZ, ZD, GW, and RL. Analysis and interpretation of data (e.g., statistical analysis): MZ, DZ, and GW. Writing, review, and/or revision of the manuscript: MZ, GW, and RL. Administrative, technical, or material support (i.e., reporting or organizing data, constructing databases): MZ, GW, and RL. Study supervision: GW and RL. All authors contributed to the article and approved the submitted version.

## Funding

This work was supported by funding from National Natural Science Foundation of China (81903666), Science and Technology Department of Yunnan Province (202101AT070301, 202002AA100007, and 2019ZF003).

## Conflict of Interest

The authors declare that the research was conducted in the absence of any commercial or financial relationships that could be construed as a potential conflict of interest.

## Publisher’s Note

All claims expressed in this article are solely those of the authors and do not necessarily represent those of their affiliated organizations, or those of the publisher, the editors and the reviewers. Any product that may be evaluated in this article, or claim that may be made by its manufacturer, is not guaranteed or endorsed by the publisher.

## References

[1] Guo Z Guozhang H Wang H Li Z Liu N. Ampelopsin Inhibits Human Glioma Through Inducing Apoptosis and Autophagy Dependent on ROS Generation and JNK Pathway. BioMed Pharmacother (2019) 116:108524. 10.1016/j.biopha.2018.12.136 31108349

[2] Kim JH Bae Kim Y Han JH Cho KG Kim SH Sheen SS. Pathologic Diagnosis of Recurrent Glioblastoma: Morphologic, Immunohistochemical, and Molecular Analysis of 20 Paired Cases. Am J Surg Pathol (2012) 36(4):620–8. 10.1097/PAS.0b013e318246040c 22441548

[3] Liu Y Shete S Hosking F Robertson L Houlston R Bondy M. Genetic Advances in Glioma: Susceptibility Genes and Networks. Curr Opin Genet Dev (2010) 20(3):239–44. 10.1016/j.gde.2010.02.001 PMC2885452 20211558

[4] Janbazian L Karamchandani J Das S. Mouse Models of Glioblastoma: Lessons Learned and Questions to be Answered. J Neurooncol (2014) 118(1):1–8. 10.1007/s11060-014-1401-x 24522719

[5] Li Y Rogoff HA Keates S Gao Y Murikipudi S Mikule K. Suppression of Cancer Relapse and Metastasis by Inhibiting Cancer Stemness. Proc Natl Acad Sci U S A (2015) 112(6):1839–44. 10.1073/pnas.1424171112 PMC4330785 25605917

[6] Codrici E Enciu AM Popescu ID Mihai S Tanase C. Glioma Stem Cells and Their Microenvironments: Providers of Challenging Therapeutic Targets. Stem Cells Int (2016) 2016:5728438. 10.1155/2016/5728438 26977157 PMC4764748

[7] Lathia JD Mack SC Mulkearns-Hubert EE Valentim CL Rich JN. Cancer Stem Cells in Glioblastoma. Genes Dev (2015) 29(12):1203–17. 10.1101/gad.261982.115 PMC4495393 26109046

[8] Beier D Schriefer B Brawanski K Hau P Weis J Schulz JB. Efficacy of Clinically Relevant Temozolomide Dosing Schemes in Glioblastoma Cancer Stem Cell Lines. J Neurooncol (2012) 109(1):45–52. 10.1007/s11060-012-0878-4 22544650

[9] Chen J Li Y Yu TS McKay RM Burns DK Kernie SG. A Restricted Cell Population Propagates Glioblastoma Growth After Chemotherapy. Nature (2012) 488(7412):522–6. 10.1038/nature11287 PMC3427400 22854781

[10] Lathia JD Gallagher J Myers JT Li M Vasanji A McLendon RE. Direct In Vivo Evidence for Tumor Propagation by Glioblastoma Cancer Stem Cells. PloS One (2011) 6(9):e24807. 10.1371/journal.pone.0024807 21961046 PMC3178553

[11] Ma Q Long W Xing C Chu J Luo M Wang HY. Cancer Stem Cells and Immunosuppressive Microenvironment in Glioma. Front Immunol (2018) 9:2924. 10.3389/fimmu.2018.02924 30619286 PMC6308128

[12] Bao S Wu Q McLendon RE Hao Y Shi Q Hjelmeland AB. Glioma Stem Cells Promote Radioresistance by Preferential Activation of the DNA Damage Response. Nature (2006) 444(7120):756–60. 10.1038/nature05236 17051156

[13] Chinese Glioma Cooperative Group (CGCG) Chinese Glioma Atlas (CGGA). Chinese Glioma Molecular Guidelines. Chin J Neurosurgery (2014) 30(5):435–44. 10.3760/cma.j.issn.1001-2346.2014.05.002

[14] Shi LS Kuo SC Sun HD Morris-Natschke SL Lee KH Wu TS. Cytotoxic Cardiac Glycosides and Coumarins From Antiaris Toxicaria. Bioorg Med Chem (2014) 22(6):1889–98. 10.1016/j.bmc.2014.01.052 PMC4334368 24582402

[15] Cerqueira AFR Almodovar VAS Neves M Tome AC. Coumarin-Tetrapyrrolic Macrocycle Conjugates: Synthesis and Applications. Molecules (2017) 22(6):994. 10.3390/molecules22060994 PMC6152750 28617340

[16] Jia C Zhang J Yu L Wang C Yang Y Rong X. Antifungal Activity of Coumarin Against Candida Albicans Is Related to Apoptosis. Front Cell Infect Microbiol (2018) 8:445. 10.3389/fcimb.2018.00445 30662877 PMC6328497

[17] Musa MA Badisa VL Latinwo LM Patterson TA Owens MA. Coumarin-Based Benzopyranone Derivatives Induced Apoptosis in Human Lung (A549) Cancer Cells. Anticancer Res (2012) 32(10):4271–6. 10.1016/j.soc.2012.07.009 23060547

[18] Mukherjee A Ghosh S Sarkar R Samanta S Ghosh S Pal M. Synthesis, Characterization and Unravelling the Molecular Interaction of New Bioactive 4-Hydroxycoumarin Derivative With Biopolymer: Insights From Spectroscopic and Theoretical Aspect. J Photochem Photobiol B (2018) 189:124–37. 10.1016/j.jphotobiol.2018.10.003 30342308

[19] Gaspar A Matos MJ Garrido J Uriarte E Borges F. Chromone: A Valid Scaffold in Medicinal Chemistry. Chem Rev (2014) 114(9):4960–92. 10.1021/cr400265z 24555663

[20] Yu H Hou Z Yang X Mou Y Guo C. Design, Synthesis, and Mechanism of Dihydroartemisinin(-)Coumarin Hybrids as Potential Anti-Neuroinflammatory Agents. Molecules (2019) 24(9):1672. 10.3390/molecules24091672 PMC6539525 31035404

[21] Lu Y Liu Y Yang C. Evaluating In Vitro DNA Damage Using Comet Assay. J Vis Exp (2017) 128):56450. 10.3791/56450 PMC5752397 29053680

[22] Yan Y Zuo X Wei D. Concise Review: Emerging Role of CD44 in Cancer Stem Cells: A Promising Biomarker and Therapeutic Target. Stem Cells Transl Med (2015) 4(9):1033–43. 10.5966/sctm.2015-0048 PMC4542874 26136504

[23] Huang RX Zhou PK. DNA Damage Response Signaling Pathways and Targets for Radiotherapy Sensitization in Cancer. Signal Transduct Target Ther (2020) 5(1):60. 10.1038/s41392-020-0150-x 32355263 PMC7192953

[24] Ki YW Park JH Lee JE Shin IC Koh HC. JNK and P38 MAPK Regulate Oxidative Stress and the Inflammatory Response in Chlorpyrifos-Induced Apoptosis. Toxicol Lett (2013) 218(3):235–45. 10.1016/j.toxlet.2013.02.003 23416140

[25] Yue J Lopez JM. Understanding MAPK Signaling Pathways in Apoptosis. Int J Mol Sci (2020) 21(7):2346. 10.3390/ijms21072346 PMC7177758 32231094

[26] Huang YH Sun Y Huang FY Li YN Wang CC Mei WL. Toxicarioside O Induces Protective Autophagy in a Sirtuin-1-Dependent Manner in Colorectal Cancer Cells. Oncotarget (2017) 8(32):52783–91. 10.18632/oncotarget.17189 PMC5581069 28881770

[27] Zhao HG Zhou SL Lin YY Wang H Dai HF Huang FY. Autophagy Plays a Protective Role Against Apoptosis Induced by Toxicarioside N Via the Akt/mTOR Pathway in Human Gastric Cancer SGC-7901 Cells. Arch Pharm Res (2018) 41(10):986–94. 10.1007/s12272-018-1049-8 29992400

[28] Gezici S Sekeroglu N. Current Perspectives in the Application of Medicinal Plants Against Cancer: Novel Therapeutic Agents. Anticancer Agents Med Chem (2019) 19(1):101–11. 10.2174/1871520619666181224121004 30582485

[29] Van Pham P. Stem Cells and Cancer Stem Cells. In: Pham PV, editor. Breast Cancer Stem Cells & Therapy Resistance. Cham: Springer International Publishing (2015). p. 5–24. 10.1007/978-3-319-22020-8_2

[30] Huang Z Cheng L Guryanova OA Wu Q Bao S. Cancer Stem Cells in Glioblastoma—Molecular Signaling and Therapeutic Targeting. Protein Cell (2010) 1(7):638–55. 10.1007/s13238-010-0078-y PMC4875273 21203936

[31] Brescia P Ortensi B Fornasari L Levi D Broggi G Pelicci G. CD133 is Essential for Glioblastoma Stem Cell Maintenance. Stem Cells (2013) 31(5):857–69. 10.1002/stem.1317 23307586

[32] Suva ML Rheinbay E Gillespie SM Patel AP Wakimoto H Rabkin SD. Reconstructing and Reprogramming the Tumor-Propagating Potential of Glioblastoma Stem-Like Cells. Cell (2014) 157(3):580–94. 10.1016/j.cell.2014.02.030 PMC4004670 24726434

[33] Chen SF Chang YC Nieh S Liu CL Yang CY Lin YS. Nonadhesive Culture System as a Model of Rapid Sphere Formation With Cancer Stem Cell Properties. PloS One (2012) 7(2):e31864. 10.1371/journal.pone.0031864 22359637 PMC3281010

[34] Gourlay CW Ayscough KR. The Actin Cytoskeleton: A Key Regulator of Apoptosis and Ageing? Nat Rev Mol Cell Biol (2005) 6(7):583–9. 10.1038/nrm1682 16072039

[35] Song W Kwak HB Lawler JM. Exercise Training Attenuates Age-Induced Changes in Apoptotic Signaling in Rat Skeletal Muscle. Antioxid Redox Signal (2006) 8(3-4):517–28. 10.1089/ars.2006.8.517 16677096

[36] Katanasaka Y Kodera Y Kitamura Y Morimoto T Tamura T Koizumi F. Epidermal Growth Factor Receptor Variant Type III Markedly Accelerates Angiogenesis and Tumor Growth Via Inducing C-Myc Mediated Angiopoietin-Like 4 Expression in Malignant Glioma. Mol Cancer (2013) 12(1):31. 10.1186/1476-4598-12-31 23617883 PMC3641008

[37] O’Neill LA Hardie DG. Metabolism of Inflammation Limited by AMPK and Pseudo-Starvation. Nature (2013) 493(7432):346–55. 10.1038/nature11862 23325217

[38] Rodriguez EF Scheithauer BW Giannini C Rynearson A Cen L Hoesley B. PI3K/AKT Pathway Alterations Are Associated With Clinically Aggressive and Histologically Anaplastic Subsets of Pilocytic Astrocytoma. Acta Neuropathol (2011) 121(3):407–20. 10.1007/s00401-010-0784-9 PMC3417062 21113787

[39] Xiang T Jia Y Sherris D Li S Wang H Lu D. Targeting the Akt/mTOR Pathway in Brca1-Deficient Cancers. Oncogene (2011) 30(21):2443–50. 10.1038/onc.2010.603 PMC3107712 21242970

[40] Fathi N Rashidi G Khodadadi A Shahi S Sharifi S. STAT3 and Apoptosis Challenges in Cancer. Int J Biol Macromol (2018) 117:993–1001. 10.1016/j.ijbiomac.2018.05.121 29782972

[41] Fresno Vara JA Casado E de Castro J Cejas P Belda-Iniesta C Gonzalez-Baron M. PI3K/Akt Signalling Pathway and Cancer. Cancer Treat Rev (2004) 30(2):193–204. 10.1016/j.ctrv.2003.07.007 15023437

[42] Guo YJ Pan WW Liu SB Shen ZF Xu Y Hu LL. ERK/MAPK Signalling Pathway and Tumorigenesis. Exp Ther Med (2020) 19(3):1997–2007. 10.3892/etm.2020.8454 32104259 PMC7027163

[43] Rezatabar S Karimian A Rameshknia V Parsian H Majidinia M Kopi TA. RAS/MAPK Signaling Functions in Oxidative Stress, DNA Damage Response and Cancer Progression. J Cell Physiol (2019) 234(9):14951–65. 10.1002/jcp.28334 30811039

[44] Qin JJ Yan L Zhang J Zhang WD. STAT3 as a Potential Therapeutic Target in Triple Negative Breast Cancer: A Systematic Review. J Exp Clin Cancer Res (2019) 38(1):195. 10.1186/s13046-019-1206-z 31088482 PMC6518732

[45] Ventura JJ Hubner A Zhang C Flavell RA Shokat KM Davis RJ. Chemical Genetic Analysis of the Time Course of Signal Transduction by JNK. Mol Cell (2006) 21(5):701–10. 10.1016/j.molcel.2006.01.018 16507367

[46] Cagnol S Chambard JC. ERK and Cell Death: Mechanisms of ERK-induced Cell Death–Apoptosis, Autophagy and Senescence. FEBS J (2010) 277(1):2–21. 10.1111/j.1742-4658.2009.07366.x 19843174

