# Supplementary material for: Anticarin β Inhibits Human Glioma Progression by Suppressing Cancer Stemness via STAT3
Source: Front Oncol. 2021 Aug 2;11:715673. doi: 10.3389/fonc.2021.715673 (PMC8366317; doi:10.3389/fonc.2021.715673)
Supplement: Supplementary file 1 [file DataSheet_1.docx]

**Supplementary materials include:**

**1. Supplementary figures**

**Figure S1. Anticarin β has no effect on proliferation, migration and invasion in normal mouse neural stem cells.**

**Figure S2. CD44 cell marker expression in T98G and U87-MG.**

**Figure S3. Anticarin β has no obvious toxicity in the major organs of mice.**

**
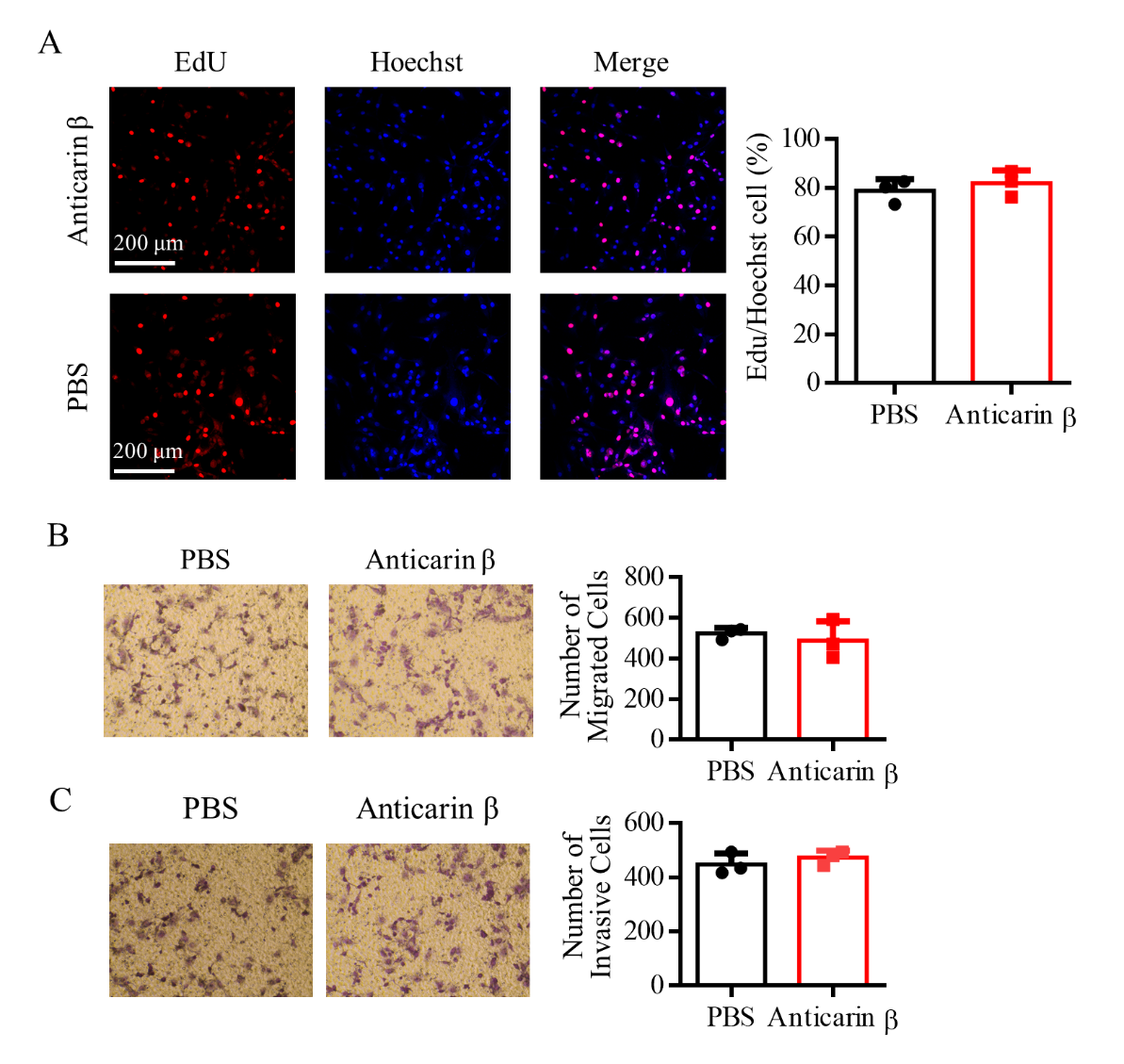
**

**Figure S1. Anticarin β has no effect on proliferation, migration and invasion in normal mouse neural stem cells.**

**(A)** The number of proliferative cells labeled by EdU (red) and cell nucleus labeled by Hoechst 33324 (blue) was different between anticarin β (0.5 μM, 12 hours) and PBS group in mouse neural stem cells. Scale bar, 200 μm. **(B)** Migration and **(C)** invasion ability of mouse neural stem cellstreated with Anticarin β (0.5 μM) for 48 hours were measured with transwell migration assay and transwell invasion assay. Data are presented as mean ± s.d. of three independent experiments conducted in duplicate, **P* < 0.05 and ***P* < 0.01, ****P* < 0.001 versus the control group.

**
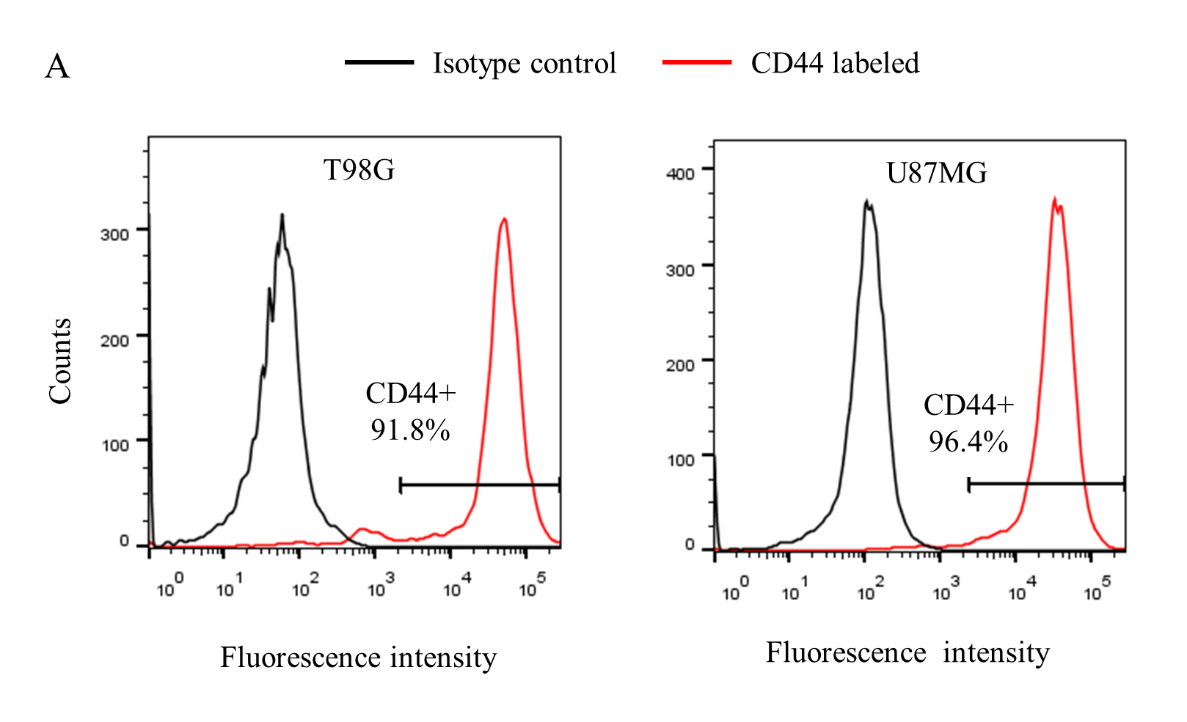
**

**Figure S2. CD44 cell marker expression in T98G and U87-MG.**

**(A)** Flow cytometric analysis of CD44+ cell marker expression in T98G and U87-MG. Glioma cancer stem cell percentage in T98G and U87-MG were evaluated by flow cytometry. Black, isotype control; Red, CD44^+^ expression. Each experiment was repeated three times.


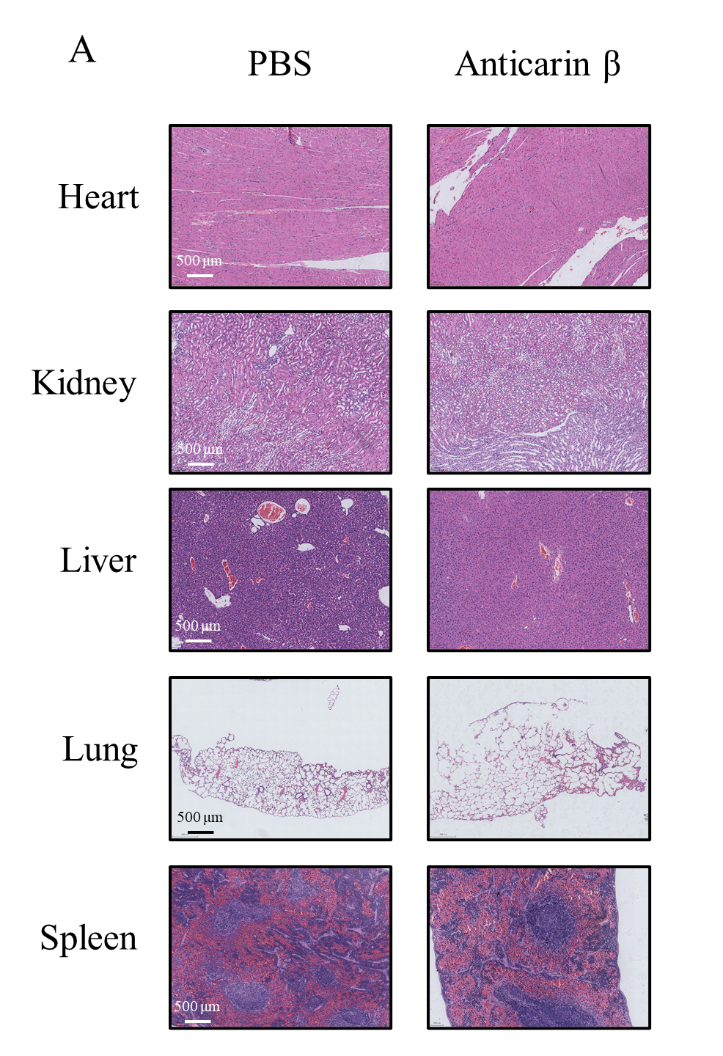


**Figure S3. Anticarin β has no obvious toxicity in the major organs of mice.**

**(A)** Critical organs (heart, kidney, liver, lungs, and spleens) were harvested, and hematoxylin-eosin staining was used to assess the systemic toxicity. Scale bar, 500 μm.
